# Virus-like Particles: Fundamentals and Biomedical Applications

**DOI:** 10.3390/ijms23158579

**Published:** 2022-08-02

**Authors:** Jorge L. Mejía-Méndez, Rafael Vazquez-Duhalt, Luis R. Hernández, Eugenio Sánchez-Arreola, Horacio Bach

**Affiliations:** 1 Departamento de Ciencias Químico Biológicas, Universidad de las Américas Puebla, Santa Catarina Mártir s/n, Cholula 72810, Puebla, Mexico; jorge.mejiamz@udlap.mx (J.L.M.-M.); luisr.hernandez@udlap.mx (L.R.H.); eugenio.sanchez@udlap.mx (E.S.-A.); 2 Centro de Nanociencias y Nanotecnología UNAM, Km 107 Carretera Tijuana-Ensenada, Ensenada 22860, Baja California, Mexico; rvd@ens.cnyn.unam.mx; 3 Department of Medicine, Division of Infectious Diseases, University of British Columbia, Vancouver, BC V6H 3Z6, Canada

**Keywords:** nanomedicine, nanotechnology, virus-like particles, preparation, characterization

## Abstract

Nanotechnology is a fast-evolving field focused on fabricating nanoscale objects for industrial, cosmetic, and therapeutic applications. Virus-like particles (VLPs) are self-assembled nanoparticles whose intrinsic properties, such as heterogeneity, and highly ordered structural organization are exploited to prepare vaccines; imaging agents; construct nanobioreactors; cancer treatment approaches; or deliver drugs, genes, and enzymes. However, depending upon the intrinsic features of the native virus from which they are produced, the therapeutic performance of VLPs can vary. This review compiles the recent scientific literature about the fundamentals of VLPs with biomedical applications. We consulted different databases to present a general scenario about viruses and how VLPs are produced in eukaryotic and prokaryotic cell lines to entrap therapeutic cargo. Moreover, the structural classification, morphology, and methods to functionalize the surface of VLPs are discussed. Finally, different characterization techniques required to examine the size, charge, aggregation, and composition of VLPs are described.

## 1. Introduction

Nanotechnology is an interdisciplinary field devoted to engineering and developing structures ranging from 1 to 500 nm. Structures that correspond to this scale are defined as nanoparticles (NPs). NPs possess promising optical, chemical, and physical properties attractive for biomedical purposes, such as diagnostic, chemical sensing, cellular imaging, drug delivery, therapeutics, and tissue engineering [1].

Given the unique physical, optical, chemical, and therapeutic properties of NPs, there has been an increasing interest in designing methods to develop and characterize them. NPs are prepared through top-down and bottom-up approaches, including laser ablation, sputtering, etching, and mechanical milling techniques. The latter encompasses spinning, chemical reduction, molecular condensation, and green synthesis processes [2]. Instead of reducing a bulk material into nanometric objects, bottom-up methods stimulate the self-assembly of atoms into bioactive NPs. For example, in nanomedicine, the amino acid side chains and functional groups of distinct proteins (e.g., collagen, elastin, gelatin, keratin, silk, and zein) are used as scaffolds to induce the self-assembly of nanofibers, nanotubes, and nanobelts to deliver drugs or develop materials for tissue engineering [3,4].

Among protein-based nanomaterials, virus-like particles (VLPs) are self-assembled platforms commercially approved by the US Food and Drug Administration (FDA) since the 1980s [5]. Since VLPs resemble the capsid morphology, structural organization, and cellular tropism of wild-type viruses [6,7], they have been exploited to prepare monodisperse nanocarriers (20–500 nm) for drug delivery, enzyme delivery for enzymatic replacement therapy [8,9], and gene therapy applications. In addition, they have been widely used to construct human vaccines against various pathogenic viruses (e.g., human papillomavirus and zika virus) and distinct types of cancer, such as colorectal, pancreatic, and cervical cancer [5,10].

In contrast to other organic NPs, VLPs are convenient, because they exhibit higher biocompatibility, the capacity for cell internalization, and ease of functionalization for cell targeting. In fact, for the latter, they can be tailored by chemical or genetic methods with various biomolecules (i.e., transferrin, folic acid, and single-chain antibodies) to enhance their bioavailability and, herein, evoke both humoral and cellular immune responses [11,12]. However, these phenomena rely on the physicochemical and biochemical characteristics of VLPs.

Nowadays, there is a constant effort to develop analytical methods that, alone or in combination with others, enable scientists to assess the influence of physicochemical parameters in the biological activities of nanomaterials. For instance, the therapeutic performance, stability, and morphology [13,14] of VLPs can vary in accord with pH ranges [15], choice of the expression system [16,17], and purification procedures [18,19].

Since the novel coronavirus SARS-CoV-2, there has been an increasing interest in reviewing VLPs as a powerful approach to producing vaccines and nanocarriers [20]. However, there is a need to complement those studies and recent ones [21], with basic principles about expressing, manipulating, functionalizing, and characterizing VLPs.

Therefore, the literature regarding the structure classification, production, morphology, and functionalization of VLPs for biomedical applications was consulted in this review. The research engines PubMed, Google Scholar, Web of Science, and Wiley Online Library databases were used to compile the literature. In addition, the same databases were used to integrate the main aspects of viruses and how the geometry is indispensable to constructing VLPs-based platforms.

## 2. Brief Description of Viruses

Viruses are entities characterized by the lack of machinery for self-replication and energy production. Their replication relies on hijacking the cellular systems of the host cells to produce the molecules necessary for their assembly and subsequent escape from the cells. Infectious viruses that have not been internalized in the host cells (not in a replication phase) and residing outside the cell host are called virions. 

Viruses are ubiquitous entities containing a DNA- or RNA-based genome protected or not by a protein shell known as a capsid and other accessory biomolecules, such as proteins and membranes [22]. The capsid shell is assembled through covalent and electrostatic interactions conferring a robust and flexible structure and made by small subunits known as capsomers [23]. These capsomers are critical components of the VLPs and constitute the basis for their self-assembly into complex structures [24]. 

The general structures of viral capsids show a diversity of conformations, including icosahedral conformation. Icosahedral capsids comprise 20 triangular subunits, whereas helical capsids are proteins assembled to form helical cylinders [25]. Despite the structural variabilities between both shapes, the functions of the viral capsid rely on the packaging, sequestering, and protection of the viral genetic material, preventing its degradation in the environment or exposure to chemical hazards [25,26]. 

Besides the capsids, viruses are surrounded or not by a viral envelope that facilitates their fusion and infection of the host cell. In addition to the presence of proteins and glycoproteins, this envelope can contain lipidic membranes acquired from the host. Viral envelopes are necessary to protect the viral genome, increase the packaging capacity, confer structural flexibility, and enable the new viruses to exit from their host cell and avert immune responses [27]. 

Viruses with envelopes are known as enveloped viruses, such as the varicella-zoster virus [28], lymphocytic choriomeningitis virus, tick-borne encephalitis virus, human immunodeficiency virus 1 [29], and severe acute respiratory syndrome coronavirus [30,31].

## 3. Key Concepts about Virus-like Particles (VLPs)

The assembly of VLPs uses exclusively viral proteins and excludes the genetic material. Thus, VLPs can be produced from a myriad of wild-type viruses, such as hepatitis B and E, tobacco, and papaya mosaic viruses [32], and from the capsids of various bacteriophages (Qβ, MS2, P22, and PP7) [33,34,35,36]. 

The production of VLPs using bacteria, mammalian cells, plants, yeast, and insect cell lines is well-documented [37,38,39]. However, current efforts are devoted to understanding their capsid self-assembly mechanisms to improve the cargo loading capacity, potency, and efficacy. 

**Figure 1:**
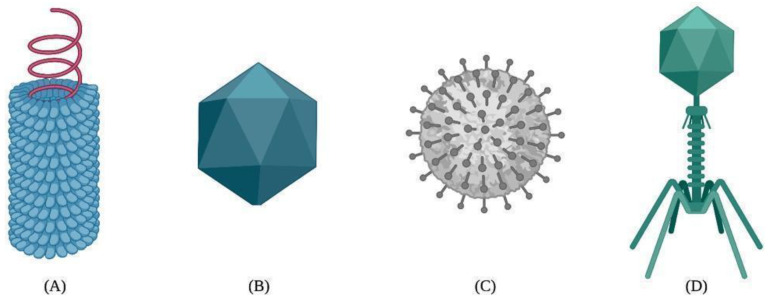
Different types of viral capsids: (**A**) helical, (**B**) icosahedral, (**C**) spherical, and (**D**) complex.

The capsid self-assembly of VLPs is a spontaneous natural process by which highly ordered structures arise from the interactions between protein monomers, also known as building blocks. The self-association between building blocks is facilitated by a thermodynamic equilibrium based on van der Waals, hydrogen bonding, hydrophobic, and electrostatic interactions during the nucleation and growth phases [40,41]. As a result, VLPs can adopt different structural arrangements, such as helical, icosahedral, spherical, or complex shapes [42,43] (Figure 1).

The self-assembly of VLPs is assisted by small molecules such as scaffolding proteins and nucleic acids [40]. In this regard, scaffolding proteins and nucleic acids can be used to aid the in vitro capsid self-assembly process of many VLPs, such as the cowpea chlorotic mottle virus, hepatitis C virus, bacteriophage MS2, simian virus 40, beak and feather disease virus, and adeno-associated virus serotype 2 VLPs [40].

Since the self-assembly of VLPs can be perturbed by changes in the salt concentration, denaturant agents, pH, and temperature [28], excipients such as polysorbate 80 are used to avoid the aggregation and preserve the stability of the VLPs [40]. On the other hand, molecules such as 2-phenoxyethanol have been used as a preservative agent to produce licensed VLP-based vaccines such as the Engerix^®^-B hepatitis B vaccine [44]. Comparably, buffering agents such as L-histidine and sodium borate have been used to manufacture the Gardasil^®^ human papillomavirus vaccine [44].

**Figure 2:**
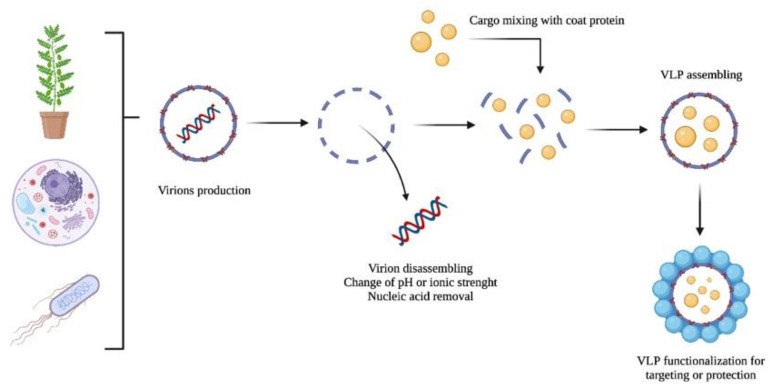
A schematic representation of the VLP production of virions as nanocarriers: (i) production, (ii) disassembling and nucleic acid removal, (iii) cargo encapsulation, and (iv) VLP functionalization.

VLP-based nanocarriers could be produced by producing viruses in their natural host, followed by virion purification, disassembling, and nucleic acid removal (see Figure 2). Then, the disassembled coat proteins are transferred into assembling conditions in the presence of any cargo to be encapsulated. Finally, the VLPs containing the load could be functionalized with any ligand or chemical moiety and covered with polymers or other compounds to reduce or enhance their immunogenicity or facilitate cell internalization. Molecules used to decorate the VLP surface include T-cell receptor ligands; polysaccharides; enzymes (e.g., α-glucosidase); and canonical amino acids (i.e., aspartic acid, cysteine, glutamic acid, lysine, and tyrosine); among others [45,46,47,48]. 

The coat proteins of the VLPs can be heterologously produced and purified from an industrial microorganism and reconstituted under suitable conditions in the presence of their cargo. In this regard, the cargo loading could be obtained by simple encapsulation when the charges of the inner portion of the VLP and the cargo surface are complementary. However, as described in detail below, the loading could also be reached by chemical or genetic modification of the coat protein on both the inner and outer surfaces.

Although most VLPs are deficient in viral genetic material, they retain properties from the native virus from which they are produced. These features include their affinity for cellular receptors, host cell entry mechanisms, and immunogenicity [49]. As native viruses, VLPs elicit both humoral and cellular responses in the host system. These responses are stimulated by VLP-based systems due to their repetitive antigenic epitopes, resembling pathogen-associated molecular patterns (PAMPs), and low polydispersity [50]. The introduction of antigens (e.g., proteins or small peptides) on the VLP surface is necessary to enhance the immune response and is achieved through chemical and genetic approaches [51,52,53]. It is worth mentioning that the immune response only occurs against the antigen, not the rest of the nanostructure produced [54].

Functionalized VLPs can trigger an immune response by activating PAMPs. These patterns are conserved molecular motifs associated with pathogen infections [55]. PAMPs are recognized by pattern–recognition receptors (PRRs), nucleotide-binding oligomerization domain-like receptors, and Toll-like receptors (TLRs) on the surface of phagocytic cells [56]. Due to the similarity of VLPs with wild-type viruses, the morphology of these NPs stimulates adaptive and innate immune responses, including cellular uptake. For example, VLPs can be taken up by enterocytes, specialized intestinal epithelial cells, dendritic cells (DCs), and macrophages [57]. This process depends upon the size, shape, and surface charge of the VLPs [58].

**Figure 3:**
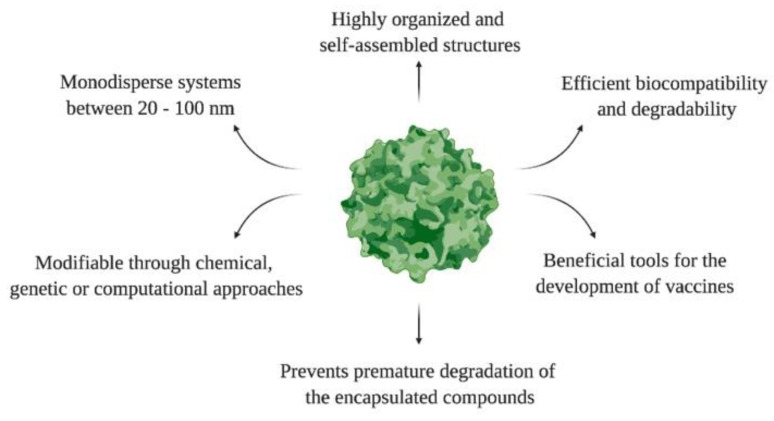
General features of VLPs.

Like other NPs, the surfaces of VLPs can be modified with various ligands to improve their therapeutic efficacy, bioavailability, and cellular interactions. The introduction of multiple molecules on the VLPs surface is known as multivalence. Then, VLPs are multivalent engineered nanoplatforms that form independent ligand–receptor bonds, elicit therapeutic responses, and mitigate endemic diseases. For example, Garg and coworkers developed a VLP-based multivalent vaccine (CJaYZ) against four arboviruses, including the zika, chikungunya, yellow fever, and Japanese encephalitis viruses. Even though the CjaYZ vaccine promoted a neutralizing antibody response against the four viruses in Balb/c mice models, further studies are required to assess the precise antigen amount of individual VLPs and their efficacy in other animal models [59]. The general features of VLPs are depicted in Figure 3.

## 4. Structure Classification of VLPs

Given the structural diversity of VLPs, they have been categorized into three main groups: enveloped, nonenveloped, and chimeric. For example, enveloped VLPs (eVLPs) are expressed using eukaryotic systems and as wild-type viruses. eVLPs are complex structures that own a host–cell-derived membrane and one or more glycoproteins. The viral envelope in eVLPs can be engineered to display heterologous adjuvants and antigens; however, this process might alter their downstream processing due to the possible presence of host cellular contaminants [60]. 

As vaccines, eVLPs stimulate immune responses and are manipulated with chemical or genetic methods. In this regard, a handful of eVLPs have been produced from pathogenic viruses for vaccine development. Some examples include eVLPs derived from the West Nile virus (WNV), dengue virus (DENV), JEV, Rift Valley fever virus (RVFV), and Ross River virus (RRV), among others [61,62]. For drug delivery applications, Rous sarcoma virus (RSV) eVLPs displaying a single-chain variable fragment (scFv) of humanized CC49 antibody (hCC_49_) have been expressed on silkworm larvae to deliver doxorubicin into human colon carcinoma cells [63]. In addition, doxorubicin was loaded into hCC49 scFv-displaying RSV VLPs by electroporation.

Non-eVLPs are single or multiple capsid protein nanoconstructs that lack cell membranes. Members of this category are produced on eukaryotic or prokaryotic expression systems. The surfaces of non-eVLPs can also be manipulated with chemical and genetic approaches to display epitopes or peptides on their surfaces and, herein, elicit wider immunological responses [64]. For instance, non-eVLPs derived from the coxsackievirus B3 antigen have enhanced humoral immune responses and protected murine models against myocarditis [65]. Additionally, rotavirus non-eVLPs (Ro-VLPs) were produced in *Nicotiana benthamiana* plants. The immunogenicity and tolerance of Ro-VLPs were evaluated in adults, toddlers, and infants [66]. 

Chimeric VLPs (cVLPs) are nanoplatforms from structural components originating from at least two different viral serotypes [67]. In these nanoplatforms, the VLP core can be modified with antigens that cannot self-assemble or present polyproteins from distinct viruses [68]. In contrast with the other two categories of VLPs, cVLPs are useful to present foreign epitopes; entrap multiple therapeutic or diagnostic molecules; and target cells, tissues, or organs [42]. However, the production of cVLPs depends upon various factors, such as the type of conjugation between proteins, glycosylation patterns, cell type, length of the fused antigen, and steric effects.

For biomedical purposes, cVLPs have been prepared from major structural components of influenza viruses (e.g., M1 protein) and human immunodeficiency virus type-1 (HIV-1) (e.g., Group-specific antigen; Gag) to target colon carcinoma cell lines and for vaccination purposes [69]. In another study, murine polyomavirus cVLPs were manipulated to elicit CD8^+^ and CD4^+^ T cells and antibody responses [70]. The immune response against other cVLPs has also been evaluated recently, specifically for the foot-and-mouth disease virus cVLP vaccine, based on the co-expression of the HIV-1 Gag protein and rabies glycoproteins [71]. 

## 5. Expression of VLPs 

Recombinant proteins are derived from the expression of recombinant DNA within living cells. Once a DNA fragment is inserted into cell lines, the cells are induced to express the protein of interest.

An expression vector is used to introduce genetic materials into cells. This vector contains sequences upstream of the cloned gene that controls its transcription and translation [72]. Non-viral expression vectors are DNA plasmids that can be delivered into the cells (transfection) as naked DNA or in association with biomaterials (e.g., polymers or cationic lipids) [73]. These vectors can be introduced to cells using photoporation, hydroporation, sonoporation, and electroporation [58]. 

On the other hand, viral vectors are optimized platforms able to transfer genetic material into host cells [74]. Various expression systems have been developed to deliver genetic material to hosts, such as *Escherichia coli*, *Bacillus subtilis*, *Pichia pastoris*, baculovirus/insect cells, plant cells, and mammalian cells [75]. For clinical purposes, recombinant proteins like interferons, growth factors, thrombolytic drugs, and hormones have been produced in *E. coli*, Chinese Hamster Ovary cells, *Saccharomyces cerevisiae*, murine myeloma cells, etc. [76]. Some of these systems have already been tried to treat diabetes, multiple sclerosis, congestive heart failure, cancer, anemia, and asthma [77]. 

For VLP production, heterologous gene expression is used to clone the gene(s) of interest (e.g., virus coat proteins). For example, the heterologous expression has been used to produce VLPs derived from the coat proteins of (1) single- and double-stranded DNA viruses (e.g., human adenovirus B (type 3), canine parvovirus, and JC polyomavirus; (2) single-stranded RNA positive-sense viruses (e.g., Lassa virus, H9N2 avian influenza virus, and Ebola virus); (3) single-stranded RNA negative-sense viruses (e.g., Flock house virus, cytomegalovirus, and papaya mosaic virus); and (4) double-stranded RNA viruses (e.g., rotavirus and infectious bursal disease virus).

## 6. Morphology and Manipulation of Viral Capsids 

**Figure 4:**
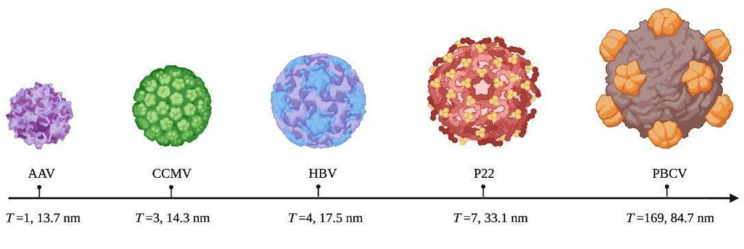
Several triangulations and diameters of viral entities. AAV, adeno-associated virus; CCMV, cowpea chlorotic mottle virus; HBV, hepatitis B virus; P22, bacteriophage; and PBCV, *Paramecium bursaria Chlorella* virus.

The Caspar-Klug theory (CK theory) dictates the symmetry of the viruses. This theory assumes that most viruses adopt icosahedral arrangements that vary in size from 20 nm to 800 nm, dictated by the number of protein–protein and protein–nucleic acid interactions [78,79]. These events promote the arrangement and maturation of the capsomers, receptor binding, cell uptake, and release of genetic material. However, the structural architecture and symmetry of viral capsids are dictated by the triangulation number (*T*). This triangulation number is important, because the higher this value is, the more the diameter of the capsid and its loading capacity and number of interactions will increase [78,80] (Figure 4). For example, the capsomers of an icosahedral capsid contain either pentagonal (pentons) or hexagonal (hexons) subunits, and the *T* number defines their symmetry and location. The *T* number is defined as the subdivision of the 20 triangular shapes that conform to an icosahedron. 

**Figure 5:**
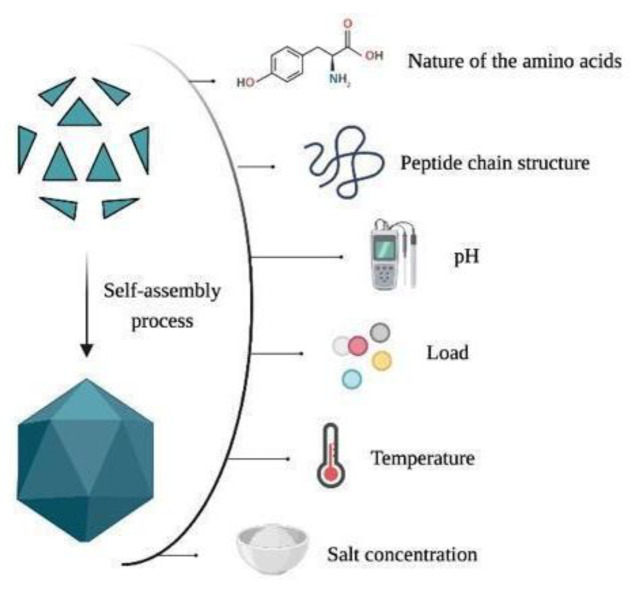
Factors influencing the design and production of VLPs.

The simplest capsomers are *T* = 1, built by 60 *T* subunits. In contrast, more complex structures result in higher *T* values. The interpretation and manipulation of these values allow the modification of the VLP diameter, rigidity, and the number of interactions between the capsid subunits [80]. Previous studies on the protein organization in capsomers demonstrated the presence of complementary proteins such as scaffold proteins (e.g., the proteins gp8, gp9, and gp10), which prevent the formation of incorrectly folded structures, stimulating growth, stability, and maturation of the capsid [81]. Moreover, other factors also affect the folded structures, such as the nature of the amino acids, the peptide chain structure, the pH, the load, the temperature, the salt concentration, and the presence of denaturing agents (see Figure 5) [82].

Likewise, it has been explained how the self-assembly of capsomers considers the formation of covalent bonds (e.g., disulphide bridges and interactions with Ca^++^), as well as weak interactions (e.g., van der Waals forces and ionic and hydrogen interactions) between the amino acid residues and the capsomers located in the structure. However, interactions may occur between the capsomers [83]. Therefore, manipulating the interactions and the other elements mentioned above can be used to improve the changes related to the resistance, stability, and hardness of the viral particles in ex vivo models [84]. 

VLPs have three available interfaces to be manipulated, either chemically or genetically. These include the external interface, the interface between protein subunits, and the internal interface. The last of these structures has been used to encapsulate materials of therapeutic interest from enzymes [85], genes [86], imaging agents [87], and DNA or RNA [88]. Thus, the molecular precision offered by the combination of nanotechnology and chemical bioconjugation techniques allows the intrastructural modification of capsomers through the insertion or extension of amino acid fragments (e.g., lysine, cysteine, aspartate, glutamate, and tyrosine residues) [89]. The result is an entirely sophisticated nanometric platform at the end of the process regarding its tropism and cell uptake [74]. 

In this regard, remarkable improvements are achieved regarding the capacity for therapeutic cargo, plasmids, mRNAs, siRNAs, antibodies, and peptides delivery [90].

For more than a decade, the design of drug delivery systems has been considered successful, especially during the encapsulation of chemotherapeutic agents from materials designed by nanotechnology [91,92]. For example, successful results were obtained when doxorubicin was entrapped upon an extensive modification of many modularized peptides (i.e., tumor-targeting peptide, lipophilic peptide NS5A1-31, and 6xhis tag) from the internal face of the VLP derived from the hepatitis B virus [93]. Likewise, studies have focused on using MS2 VLPs modified with the SP_94_ peptide to release siRNA cocktails (<150 pM); ricin toxin A-chain (RTA) (100 fM); and chemotherapeutic drugs such as doxorubicin, cisplatin, and 5-fluorouracil (<1 nM) into human hepatocellular carcinoma and human epidermoid carcinoma cell lines [94].

## 7. Functionalization of the VLPs 

**Figure 6:**
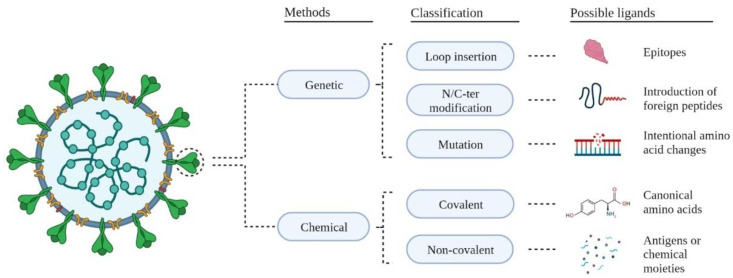
Methods, classification, and possible ligands to functionalize VLPs.

The surface modification of VLPs can be achieved with covalent, noncovalent, and genetic approaches. Figure 6 illustrates this classification and possible ligands used to functionalize VLPs. In the covalent approach, canonical amino acids (i.e., aspartic acid, cysteine, glutamic acid, lysine, and tyrosine) are incorporated to act as reactive side chain moieties able to form biocompatible bonds with the VLP surface [48]. In the case of noncovalent methods, electrostatic interactions include antigens and adaptors to decorate the surfaces of VLPs [75]; however, these approaches might lead to unstable VLPs during storage.

In genetic procedures, small or entire proteins are accommodated on the VLP surface via loop insertion, N-terminus/C-terminus (N/C-ter) modification, or the mutation of amino acid residues. In loop insertion approaches, epitopes are introduced into the surface-exposed loops of capsid proteins from VLPs to induce strong neutralizing responses [95]. During N/C-ter modification, foreign peptides are introduced into the N/C-ter of the capsid proteins from VLPs without altering their immunogenicity or structure; this approach is commonly implemented during cVLP production (see Figure 6) [95]. The mutation of amino acids has been performed to add chemical reactivity to specific sites of the VLP surfaces, modify their immunogenicity [47], and evaluate their self-assembly and stability [96]. In this regard, genetic methods have been used to redesign the surfaces of VLPs with drug delivery and in vivo imaging applications [97]. Nevertheless, such approaches have also helped construct VLPs to treat lysosomal storage diseases [47].

Post-translational modifications (PTMs) are biochemical events that change the properties of a protein after translation [78]. In such biomolecules, PTMs occur from enzymatically adding groups to one or more specific amino acids in the protein. The chemical moieties added during this process include acetyl, glycosyl, phosphoryl, methyl groups, etc. [98]. Although incorporating such molecules might compromise the activity, physicochemical properties, conformation, stability, and localization of proteins [99], they might also increase the pharmacological properties of peptides and drive their proper folding into three-dimensional structures [100,101].

For VLPs, PTMs depend upon the expression system used and are convenient for enhancing their immunogenicity, antigen stability, and therapeutic properties. For example, a Q*β* bacteriophage VLP-based vaccine produced in *E. coli* has been phosphorylated at Thr^181^ to reduce the aggregation of hyperphosphorylated pathological Tau (pTau) in non-Tg and rTg4510 mice [102]. In another study, VLPs vaccines expressed on *Nicotiana benthamiana* displayed influenza hemagglutinin (HA) glycoproteins and elicited immunoglobulin G (IgG) and immunoglobulin E (IgE) responses in 34% of subjects without hypersensitivity or allergic reactions [103]. 

In contrast, baculovirus expression systems (BES) have permitted other PTMs on VLPs, such as acylation, mannosylation, and disulphide bond formation. Such modifications have enabled the expression of multiple VLPs sharing functional similarity, structural arrangements, and antitumor response enhancement [104,105]. In plant expression systems, the stability and proper folding of VLPs are attained with *N*- and *O*-glycosylation patterns. These PTMs are entailed during cell adhesion, protein targeting, and immune responses [106]. 

## 8. Characterization

Considering that VLPs are NPs, we can characterize them following the standard techniques used to reveal the morphology, size distribution, zeta potential (ζ-potential), molecular weight (*M*_w_), and elemental composition of NPs [107]. Examining such features is relevant to promoting their interaction with cells, designing nanoplatforms for biomedical applications, or averting their toxicity [108]. 

The particle morphology of VLPs can be examined with transmission electron microscopy (TEM) and scanning electron microscopy (SEM). However, other types of electron microscopy (EM), such as cryo-EM, have been used to report the morphology of influenza HA VLPs and visualize their interactions with murine DCs [109].

For VLP-based vaccines, TEM has been used to confirm the size and morphology of many VLP-based vaccines, for example, spherical porcine encephalomyocarditis virus (EMCV) VLPs (30–40 nm) [110], HPV VLPs (40–60 nm) [111], and mutated *Bombyx mori* cytoplasmic polyhedrosis virus VLPs (50–70 nm) [112].

In the case of SEM, the size, shape, and surface composition can be analyzed. For VLPs, this tool has been applied in combination with TEM to observe the size and corroborate the morphology of SARS-CoV-2 VLPs [113], influenza H7N9 VLPs (120 nm) [114], and *Macrobrachium rosenbergii* nodavirus VLPs (27-30 nm) targeting epidermal growth factor receptor (EGFR)-positive colorectal cancer cells [115]. In addition, the distribution of the sizes of NPs is complemented with other characterization techniques such as dynamic light scattering (DLS). 

DLS, also known as photon correlation spectroscopy (PCS) or quasi-elastic light scattering (QLS), is a noninvasive tool used to measure the Brownian motion of macromolecules in a solution [115]. A DLS analysis has been used to study NP size distribution—from submicron to nanometers—and detect conformational changes of nucleic acids or to study the sizes of various nanoformulations [115,116,117]. This method is widely used for protein-based nanomaterials to detect aggregates in macromolecular solutions, the size of proteins, or to monitor the binding capacity of ligands [118]. For VLPs, DLS has been established as a simple method suitable for uncovering the size and aggregation of VLPs. For example, it has been used as a powerful method to detect the aggregation of VLP-based vaccines, such as quadrivalent HPV VLPs [119]. In combination with other characterization techniques, such as circular dichroism (CD) and UV–Vis spectroscopy, DLS has been used to monitor the hydrodynamic size and stability of Norwalk virus (NV) VLPs at variable temperatures and pH conditions [120,121]. 

DLS is also used to determine the ζ-potential of NPs. The ζ-potential, also known as the electrokinetic potential, is the potential at a colloid particle’s slipping/shear plane moving under an electric field. The ζ-potential reflects the potential difference between the electric double layer of electrophoretically mobile particles and the dispersant layer around them at the slipping plane. The magnitude of the ζ-potential indicates the degree of electrostatic repulsion between adjacent, similarly charged particles in a dispersion. Thus, the ζ-potential is an important indicator of the stability of colloidal dispersions. A high ζ-potential value (+ or −30 mV) will confer stability, and the solution or dispersion will resist aggregation. This value is affected and could be modulated by the VLP functionalization.

To assess the magnitude of the ζ-potential, DLS instruments require minimal sample preparation and low-cost laboratory materials. DLS instruments are provided with a laser, light detector, and sample holder. For ζ-potential measurements, particles suspended in the medium scatter the incident laser light in all directions, and hence, the scattering intensity is recorded by the detector [115]. Since the frequency between the scatter and original light are different, they are optically mixed, deduced from the Doppler shift, and the ζ-potential is calculated by different equations [122].

In terms of the sample analysis, DLS measurement conditions must be defined to avoid variabilities on the resultant ζ-potential; the factors that influence this value are temperature, solvent ratio (e.g., organic solvents), pH, presence or not of surfactants, the existence of ions in a solution, and ionic strength [123,124,125]. In addition, the DLS analysis must consider the intrinsic features of the produced NPs to report the acquired ζ-potential; these characteristics include their size, shape, sedimentation, polydispersity, and presence of conjugates [126,127]. 

For VLPs, DLS instruments have been used to record the ζ-potential of various preparations, such as VLPs derived from GI.1 and GII.4 noroviruses and the feline calicivirus at different pHs, temperatures, and ionic strengths [128]. In another study, the ζ-potential of the Physalis mottle virus (PhMV) VLPs used as nanocarriers of photosensitizers (i.e., Zn-EpPor) and drugs (i.e., doxorubicin and mitoxantrone) was studied using DLS [87].

CD remains one of the gold standards for evaluating the secondary structure, intermolecular interactions, folding, ligand-binding properties, and stability of proteins [129]. The structural behavior of such biomolecules is reflected in the CD spectra, which are divided into two regions. The first region is referred to as far-ultraviolet (UV) (190–250 nm), and the second one is termed the near-UV (250–320 nm) region [130]. In this sense, the former is used to characterize protein secondary structures, and the latter provides an insight into the tertiary structure of various proteins [131]. 

Despite the differences between such regions, both have been useful in understanding the stability of protein structures against variable conditions. For VLPs, the CD has been performed to evaluate the influence of temperature, pH, and ionic strength on the stability of VLPs derived from the noroviruses GI.1 and GII.4 [132]. Another study has used it to estimate the secondary structure (*α* and *β*-sheet contents) and thermal stability at 52 °C of prawn nodavirus capsid VLPs displaying the HBV ‘a’ determinant [133]. However, other spectroscopic techniques, such as UV–Vis spectroscopy and nuclear magnetic resonance (NMR), are also recommended.

UV–Vis spectroscopy is another absorption spectroscopy technique used to characterize organic and inorganic samples. In this method, the absorption of UV–visible radiation causes the excitation of electrons from lower to higher energy levels and is based on the Beer–Lambert law. In proteinaceous samples, the UV absorption occurs from 180 to 230 nm due to $\pi \to \pi^{*}$ transitions in the peptide bonds; this phenomenon is based on the capacity of aromatic side chains (tryptophan, tyrosine, and phenylalanine) to absorb UV–Vis radiation [134]. For the characterization of NPs, UV–Vis spectroscopy is a suitable method because of its optical features that are sensitive to concentration, size, shape, and agglomeration [135]. For VLPs, this tool has been used to evaluate the enzymatic activity and aggregation index of glutathione produced on P22 VLP nanoreactors [136]. In addition, UV–Vis spectroscopy has been employed to detect the conjugation ratio and confirm the functionalization with a glycosylated mucin-1 peptide of breast cancer VLP-based vaccines derived from RHDV [137]. 

As aforementioned, NMR spectroscopy is used during the characterization of VLPs. NMR is a reproducible and nondestructive technique that describes the response of nuclei (i.e., ^1^H, ^13^C, ^5^N, and ^31^P) to an applied magnetic field [138]. At an atomic resolution level, the NMR spectroscopy analysis yields information about protein complex interactions, conformational changes, self-assembly, and thermodynamics in near-physiological conditions [139,140]. For NPs, this technique is used to examine their formation, morphology, and physical properties in solution or solid states [141]. In the case of VLPs, NMR spectroscopy has been used to monitor the disassembly process of Q*β*-VLPs labeled with ^19^F in cell lysates and combined with microscopy techniques (i.e., confocal fluorescence microscopy) to observe their cellular internalization [142]. However, this event can be influenced by the *M*_w_ of such nanoplatforms.

The *M*_w_ of polymeric NPs influences their cargo capacity, the release profile of therapeutic molecules, and the cell internalization process [143]. There are multiple methods to separate NPs through their *M*_w_. For instance, the size and charge by gel electrophoresis, sodium dodecyl sulfate-polyacrylamide gel electrophoresis (SDS-PAGE), capillary electrophoresis (CPE), isoelectric focusing, etc. [144]. Electrophoresis is performed to separate complex protein samples from cells and immunoprecipitate, column fractions or subcellular fractions [145]. In this method, two electrodes of opposite charges connected by a supporting medium (e.g., cellulose acetate, agarose, starch gel, and polyacrylamide gel) are used to induce the migration and separation of charged particles.

For protein-based NPs, some of these methods have been useful in assessing the *M*_w_ and purity of NPs directed at the tumor microenvironment [146]. For VLP separation, CGE has been manipulated to characterize the process and formulation of the component protein sizes and ratios of VLP-based vaccines derived from Western, Eastern, and Venezuelan equine encephalitis (WEVEE) viruses [147]. Moreover, microfluidic gel electrophoresis has been used to characterize Q*β*-VLP mutants (i.e., K16F and K16Y), who manifested varying degrees of self-assembly and interactions with plasma membrane components [148]. The *M*_w_ of VLPs is inspected with other analytical techniques such as charge detection mass spectrometry (CDMS).

CDMS is a quantitative method that provides information about the *M*_w_ of DNA fragments, natural products, peptides, and proteins [149]. This technique is based on producing ion fragments separated according to their mass-to-charge ratio (*m*/*z*) [150]. For the interested reader, the fundamentals of this tool, types, and potential applications have already been reviewed by Glish and Vachet [151]. For NP characterization, CDMS contributes to understanding their elemental, molecular, chemical state, and structural information. Therefore, it has been applied to characterize various nanostructures, such as metal nanoclusters containing gold or silver [152,153]. For VLPs, some modalities of MS, such as native electrospray ionization MS, have been used to assess the *M*_w_ of VLPs derived from Norovirus West Chester GI.1., CPMV, and bacteriophages P22 and T5 [154]. In another study, charge detection MS (CDMS) has been mentioned as a robust method that can provide the mass distribution of antigens, their masses, and the presence of impurities on VLP-based vaccines [155].

## 9. Discussion

In nature, proteins participate as catalysts, cellular and molecular processes mediators, and building blocks in eukaryotic and prokaryotic living forms. In biotechnological research, proteins are used to manufacture novel molecules (e.g., antibodies, hormones, and enzymes) able to modulate immune responses, diagnose diseases, and deliver therapeutic compounds [156]. Given their functions, proteins can be used to design biodegradable, stable, safe, and effective nanomaterials with therapeutic activities [157].

VLPs are a special class of protein-based nanomaterials that resemble the architecture and symmetry of wild-type viruses. As mentioned in previous sections, the symmetry of VLPs is dictated by their *T* number; the larger this number is, the larger their size and cargo capacity (see Figure 4). This parameter is controlled by modulating the media conditions, such as pH and salt concentration. It is of great importance to understand, as it can be manipulated to display epitopes, improve the packaging capacity of bioactive molecules, and enhance the interaction of VLPs with immune cells such as B cells [158].

Another interesting feature of VLPs is that they are expressed in eukaryotic and prokaryotic cells. Table 1 denotes that expression systems, such as bacteria strains, plants, mammalian cells, and insect cells, have been used to produce VLP-based vaccines against cancer or viral infections. Each system is selected based on its cost, reproducibility, scalability, and purpose of the study. In addition, their advantages and disadvantages are key aspects to consider. For example, bacteria cells (e.g., *E. coli* strains) are preferred because of their growth rate, high expression yield, and ease of scaling-up. Similarly, the use of other expression systems such as yeast cells (e.g., *S. cerevisiae*, *P. pastoris*, and *H. polymorpha*) can be scalable, cost-effective, and intrinsically, they functionalize (partially) VLPs by PTMs [159,160]. In contrast to bacteria expression systems, mammalian cells are not cost-effective, but they are special, as they can be used to express eVLPs and non-eVLPs for the mass production of glycosylated products [160,161].

Regarding the use of plants to produce these platforms, they have been reviewed as a low-cost scalable system to express well-assembled VLPs in high quantities [165]. However, given the capacity of plant cells to decorate the surfaces of VLPs with distinct glycoforms at different patterns, the produced VLPs can induce diverse side effects [166]. Finally, insect cells (e.g., Sf9 or High Five^TM^) can contribute significantly to VLP production because of their high protein expression levels and capacity to express numerous proteins simultaneously, which is convenient for constructing eVLPs, non-eVLPs, and cVLPs for vaccine development [167]. However, the presence of host–cellular contaminants can be laborious to remove and may interfere with the interaction between the produced VLPs and their cellular targets; this event is translated into lower immune responses.

In addition to the processes from which eukaryotic or prokaryotic cells express the coat proteins of VLPs, the heterologous expression of the coat proteins allows genetic manipulation to improve the VLP properties, such as drug delivery [168,169], enzyme delivery for enzymatic replacement therapy [8,9], enzymatic nanoreactors [15,170], nanoreactors for drug activation [171,172], immunotherapy for cancer treatment [168,173], and medical imagenology [87,174]. Interestingly, no information on using VLPs to entrap bioactive compounds such as natural products could be found in the literature. Thus, this area must be explored to further strengthen the applications of VLPs.

Functionalization procedures are the cornerstone to enhancing the drug release, interaction with cells, and efficacy of NPs. According to our review, the surfaces of VLPs are decorated with genetic and chemical methods. The former is applied to construct VLPs as nanoreactors or drug delivery systems, whereas the latter is implemented to fabricate VLPs as nanocarriers of genetic material, drugs, magnetic resonance imaging (MRI) agents, photosensitizers, vaccines, and NPs [48,175]. However, their implementation could represent some disadvantages that are noteworthy to mention.

For instance, genetic methods must consider the size of the biomolecule to be inserted, as it can induce detrimental effects on the assembly and stability. In addition, the resemblance of the VLP-based preparation to the native virus from which it was produced can be disrupted [176]. On the other hand, chemical methods are unspecific and can be expensive and require complex purification procedures. Furthermore, the successful incorporation of chemical ligands can be difficult to analyze, since they might not be completely incorporated in VLP surfaces [177,178]. Even though these differences can impede VLP applications, the successful incorporation of ligands and their effects on VLP morphology, size, and composition must be determined by characterization methods.

In nanomedicine, the characterization of nanomaterials is a major concern required to understand their interaction and fate in biological systems and their safety and therapeutic efficacy [179]. Features such as the size, shape, and biochemical properties of NPs can vary against methodological (i.e., synthesis method), environmental (i.e., storage time), and commercial (i.e., laboratory supplier) factors [180].

VLP-based preparations are rigorously characterized by several analytical techniques commonly used in inorganic nanotechnology and protein chemistry. For vaccine development, the size of VLPs is a crucial first step to decide, since this parameter influences their interaction with immune cells and, hence, the immune response that VLPs provoke. Comparably, controlling their geometry and molecular patterns by modifying the temperature, pH, and salt concentration can result in nanoplatforms with different capacities to induce humoral immune responses and side effects [181].

In view of the importance of the size, shape, and arrangement of VLPs, we reviewed the fundamentals and applications of DLS, TEM, UV–Vis, CD, MS, and NMR techniques to study such parameters, as well as their composition, *M*_w_, and assembly. 

For NP characterization, spectroscopy techniques such as DLS allow the detection of agglomerates, size analysis, and yield information about the possible shape of the produced nanoplatform. This method is extremely advantageous, since it is easy to perform for homogeneous samples, cost-effective, and does not require elaborate sample preparation procedures. However, its use can represent significant limitations; for instance, samples must undergo constant Brownian motion during the analysis to avoid variabilities throughout data acquisition. Moreover, since numerous calculations are performed in the interface of DLS instruments, various assumptions are made regarding the shape of the NPs, which usually is assumed to be spherical [107]. Therefore, the morphological analysis of the nanomaterials must be complemented with TEM. 

Since the development of TEM in the 1930s, it has been a powerful technique widely used to examine, individually, the qualitative features of many biological samples and NPs at a high-resolution capacity [182]. However, special training is needed to manage its operating system and complex sample preparation methodologies. In addition, it has been reviewed that the sample drying process is critical and that, if TEM is not operated carefully, the sample can be degraded at high voltages [183].

Regarding the use of other visualization techniques, new protocols for cryogenic electron microscopy (Cryo-EM) can be advantageous in determining the actual structure of VLPs in aqueous systems [184]. Facilitated by recent advances, Cryo-EM has become a powerful tool to routinely solve near-atomic resolution three-dimensional protein structures preserved by embedding them in an environment of vitreous water. However, since biological samples (e.g., viral proteins, organelles, and tissues) are sensitive to radiation, and this technique can lead to poor image quality due to sample heterogenicity [185].

On the other hand, when developing specific protocols for analyzing VLP-based preparations by liquid chromatography-tandem mass spectrometry (LC-MS/MS), this technique is a versatile, robust, and sensitive methodology used to characterize many protein molecules [186]. However, the use of MS can be limited by the ionization process, incomplete ionization, and interference. These problems can be resolved by considering other ionization techniques, such as time-of-flight MS (TOF-MS) or ion capture MS. Developing unique strategies can improve the precision, accuracy, and sensitivity of MS detection. 

According to the experience of our group in NMR analyses [187,188], we consider that their use in NP characterization must be strengthened in certain aspects. For example, even though NMR is a distinguished laboratory technique among organic chemists, its utilization requires training and expertise in data curation, especially for scientists who actively participate in nanomedicine research fields [141]. In addition, knowledge about instrumentation and sample preparation before a NMR analysis must be mandatory.

Nowadays, there are excellent reports regarding the production of VLPs. However, we consider that this work complements the importance of considering additional factors (i.e., *T* number, limitations of characterization techniques, and large-scale application) to use VLPs as vaccines or nanocarriers for therapeutic applications. Moreover, for the interested reader, this review provides insight into the basic principles of the common characterization techniques used in nanomedicine. Therefore, this work can be followed not only by specialists but also by students involved in the biomedical field.

## 10. Conclusions

This review discusses the VLPs as self-assembled sophisticated nanometric systems resembling wild-type virus structural and biological properties, showing great potential in the biomedical field. VLPs are constituted by different structural components that make them suitable to present antigens or deliver drugs, genes, or imaging agents into experimental models.

This work compiled the recent scientific literature about expressing VLPs in different organisms and cell lines to prepare vaccines or nanocarriers for biomedical purposes. In addition, an insight into other analytical techniques to assess the chemical and physical composition of VLPs derived from various viruses is provided. However, in contrast to conventional protocols for nanosized materials and protein chemistry, new and specific protocols for VLP characterization should be developed. Finally, new analytical techniques could be established to improve the VLP-based preparations to understand better and modulate the encapsidation processes.

Further studies must assess their safety, stability, integrity, potential toxicity, and medical potency against variable temperatures and pHs.

## Figures and Tables

**Table 1:** VLPs with biomedical applications: expression systems, structure, and features.

Name	Expression System	Shape	Features and Biomedical Applications	Reference
tHBcAg VLPs		Icosahedral 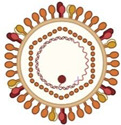	Encapsidates a plasmid that codes for a short hairpin RNA fragment. Targets anti-apoptotic *Bcl*-2 gene. Uses a truncated hepatitis B core antigen. Folic acid was conjugated to target the folate receptor on HeLa cells.	
	*E. coli* strain W3110IQ			[162]
SARS-CoV-2 VLPs		Corona-like 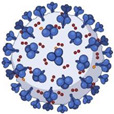	The membrane (M) and envelope (E) proteins enabled the formation and promoted the release of SARS-CoV-2 VLPs. The use of distinct cell lines caused differences in the size and shape of the produced VLPs. These constructs could be use in vaccinology against COVID-19 and virus research.	
	HEK-293T and Vero E6 cells			[161]
HIV-1 Gag-eGFP VLPs		Not reported	Given HIV-1 Gag VLPs architecture, they are considered as robust prospects for multivalent vaccines production. Sf9 cell pools were adapted to produce, on a large scale, HIV-1 Gag-eGFP VLPs. The method developed in this reference can be adapted to other VLP-based preparations to target viral diseases such as influenza and COVID-19.	
	Sf9 cells	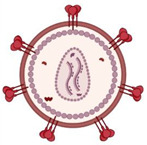		[163]
HBcAg-wDIII VLPs		Spherical	Like other Flaviviruses, wDIII induces both protective immunity and neutralizing antibody responses. *N. benthamiana* leaves yield ~95 homogeneous VLPs. 25 μg HBcAg-wDIII VLPs elicited immunological responses in BALB/c mice. The produce nanoplatform is low-cost and effective against infections caused by WNV.	
	*Nicotiana benthamiana*	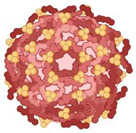		[164]

Abbreviations: VLPs, virus-like particles; tHBc, truncated hepatitis B core antigen; SARS-CoV-2, severe acute respiratory syndrome coronavirus 2; COVID-19, coronavirus infection disease 2019; HIV-1, human immunodeficiency virus serotype-1; eGFP, enhanced green fluorescent protein; WNV, West Nile Virus; wDIII, domain III of the WNV.

## Data Availability

Not applicable.
